# Congenital Cytomegalovirus Infection Manifesting as Neonatal Persistent Pulmonary Hypertension: Report of Two Cases

**DOI:** 10.1155/2011/293285

**Published:** 2011-06-12

**Authors:** Elizabeth Walter-Nicolet, Magali Leblanc, Marianne Leruez-Ville, Philippe Hubert, Delphine Mitanchez

**Affiliations:** ^1^Service de Néonatologie, Hôpital Armand Trousseau, AP-HP, Faculté de Médecine, Université Pierre et Marie Curie, 75012 Paris, France; ^2^Service de Réanimation Néonatale, Hôpital Universitaire D'Angers, 49033 Angers, France; ^3^Laboratoire de Virologie, Hôpital Necker-Enfants Malades, AP-HP, Faculté de Médecine, Université Paris-Descartes, 75015 Paris, France; ^4^Service de Réanimation Polyvalente Pédiatrique et Néonatale, Hôpital Necker-Enfants Malades, AP-HP, Faculté de Médecine, Université Paris-Descartes, 75015 Paris, France

## Abstract

Various neonatal symptoms can lead to a diagnosis of congenital CMV infection. We report two cases of persistent pulmonary hypertension in relation with congenital CMV infection following maternal primary infection and reinfection, respectively. Both infants had severe refractory hypoxemia, requiring high-frequency ventilation, inhaled nitric oxide and inotropic support. One of them required extracorporeal membrane oxygenation for five days. Ganciclovir therapy was attempted in the two cases on day 12 postnatal. One of the infant died on day 15 postnatal. The other survived and is developing uneventfully at 15 months of age. *Conclusion*: Neonatal persistent pulmonary hypertension can be the consequence of congenital CMV infection. Intensive respiratory support and IV ganciclovir are indicated in case of life-threatening condition.

## 1. Introduction

Cytomegalovirus (CMV) is one of the most common causes of congenital infection in the developed countries, affecting approximately 1% of all live births. Most of infected infants will remain asymptomatic, but about 10% of infected newborns will have symptomatic disease and 10–15% will develop problems during the first 6 years of life [1]. Symptoms include growth restriction, microcephaly, seizures, cerebral ventriculomegaly, chorioretinis, hepatitis syndrome, thrombocytopenia, anemia, and pneumonitis [2]. Preconceptional maternal immunity does not provide complete protection to the fetus, and congenital CMV infection may occur in infants of mother who are seropositive for CMV prior to pregnancy. The outcome of recurrent maternal infection may be severe [3, 4]. The benefit of antiviral therapy in congenital CMV infection is still controversial [5, 6]. We present two cases of congenital CMV disease presenting with severe persistent pulmonary hypertension (PPH).

**Case 1**

A 30-year-old woman in her second normal pregnancy delivered vaginally a 2940 g (25th percentile) female infant with a head circumference of 34 cm (50th percentile) at 39 weeks gestation. Maternal asthenia and fever complicated the antenatal course at 31-week gestation. No prenatal abnormal ultrasonographic finding was recorded. Apgar score was 10 at 1 and 5 minutes. Mechanical ventilation was initiated in the first hour of life for acute respiratory distress. Chest X-Ray showed alveolo-interstitial pneumonitis. Antibiotic therapy including amoxicillin, cefotaxime, and amikacin was initiated. Despite mechanical ventilation, refractory hypoxemia developed within 12 hours of life. Echocardiogram showed a structurally normal heart and diagnosed pulmonary hypertension (peak velocity of tricuspid regurgitation at 4 m/sec, pure right-to-left shunt at both atrial and ductal level, and reduced mean pulmonary blood velocity at 0.19 m/sec). Despite high-frequency ventilation (HFV), inhaled nitric oxide (NO) (20 ppm), inotropic support (dobutamine 10 *μ*g/kg/min), and surfactant therapy (Curosurf 200 mg/kg), the infant's condition deteriorated. Hepatomegaly and splenomegaly were noted on day 5. Biological abnormalities included elevated C-reactive protein (80 mg/L), thrombocytopenia (61 × 10^9^/L), neutropenia (0.37 × 10^9^/L), elevated conjugated bilirubinemia (total serum bilirubin 285 *μ*mol/L; direct bilirubin 185 *μ*mol/L), and aspartate aminotransferase (207 UI/L). Cultures for bacteria and fungi were negative. CMV was isolated in the urines and in the blood plasma by PCR performed on day 12. Intravenous ganciclovir treatment (12 mg/kg/d) and polyclonal immunoglobulin G (Octagam 1 g/kg every 24 hours for 2 days) were initiated. Severe hypoxemia persisted with progressive hypercapnia. Chest X-ray found severe pulmonary interstitial emphysema. The patient died on day 15. Parents declined postmortem examination and percutaneous lung biopsy. Maternal seroconversion during the seventh month of pregnancy was confirmed by retrospective analysis of maternal serum sample taken monthly for toxoplasmosis testing (IgG < 0.4 UI/l and IgM < 0.9 UI/l on the 7th month of gestation and IgG = 0.7 UI/l and IgM = 2.9 UI/l one month latter) with ETI-CMV-G and ETI-CMV-M assays (DiaSorin, France).

**Case 2**

A 33-year-old woman in her second normal pregnancy delivered vaginally a 2520 g (20th percentile) male infant, with a head circumference of 32 cm (10th percentile) at 37 weeks gestation. Apgar score was of 7 and 8 at 1 and 5 minutes, respectively, and the infant required mechanical ventilation for persistent cyanosis. Chest radiographs showed moderate alveolar syndrome. Echocardiography confirmed an anatomically normal heart and pulmonary hypertension (pure right-to-left shunt at both atrial and ductal level, hypertrophy of right ventricle, and intermittent tricuspid regurgitation). Blood cell count showed marked thrombocytopenia (20 × 10^9^/L) with normal leucocytes count. Amoxicillin and gentamicin therapy was initiated.

Despite HFV, adjunction of NO (20 ppm), and inotropic support (dobutamine 20 *γ*/kg/min, epinephrine 0.5 *γ*/kg/min), PPH worsened and was associated with refractory hypoxemia. The infant required extracorporeal membrane oxygenation support for 5 days. He tolerated the withdrawal of NO on day 4 and weaning of ECMO on day 8. FiO2 was decreased to 50%. Echocardiogram revealed infrasystemic pulmonary hypertension and moderate hypertrophy of the right ventricle. After transient respiratory improvement, echocardiogram confirmed severe exacerbation of PPH. HFV, 100% FiO2, adjunction of NO (20 ppm), and pulmonary vasoactive agent (prostacyclin 40 ng/kg/min) were required.

Blood culture and gastric swab were negative for bacteria and fungi. Because of neonatal thrombocytopenia, urine was screened for cytomegalovirus (CMV) on day 1 and confirmed congenital CMV infection (17 500 copies/ml). CMV DNA was detected by PCR in blood sample and broncho-alveolar lavage (BAL) on day 12 (resp., 500 copies/mL and 1520 copies/mL). Simultaneously, the patient presented jaundice and hepatosplenomegaly. Conjugated hyperbilirubinemia (total serum bilirubin 101 *μ*mol/L; direct bilirubin 73 *μ*mol/L) was noted. Platelet count was normal (238 × 10^9^/L). Intravenous ganciclovir (10 mg/kg/d) was initiated on day 13 and prolonged for 6 weeks with no side effect. CMV DNA amplification became negative in broncho-alveolar lavage, blood, and urine within two weeks, and the respiratory status slowly improved. NO was progressively decreased until withdrawal at day 25. The infant was successfully weaned of the ventilator on day 32, and oxygen therapy was needed until day 56. Cholestasis progressively resolved. Neurological status remained normal. Ophthalmologic examination ruled out CMV retinitis. Head CT scan showed bilateral subependymal cysts without calcifications. The infant was discharged at 2 months. Echocardiogram was normal from the 3rd month of life and remained normal at 9 months. Clinical and neurological examination at 15 months was normal. Auditory brainstem evoked responses were normal at 10-month followup.

Positive CMV-specific IgG (46 U/mL) of high avidity (96%) (VIDAS CMV IgG, BioMérieux, France) and negative IgM (VIDAS CMV IgGM) detected on maternal serum collected at 20 weeks of gestation were in favour of preexisting immunity. CMV-specific IgG antibody levels were comparable between the serum sample collected at 29-week gestation and at birth.

## 2. Discussion

We report two cases of congenital cytomegalovirus infection presenting at birth with PPH and refractory hypoxemia. Neonatal PPH is usually associated with perinatal asphyxia, meconium aspiration syndrome, hyaline membrane disease, or bacterial infection. These causes were excluded in these two cases, and PPH was most probably a direct complication of CMV congenital pneumonitis. Pneumonitis is rarely the only manifestation of congenital CMV disease in neonate [7] and its incidence is low [8]. To our knowledge, only one case of congenital CMV infection presenting as severe PPH was previously reported [9]. 

CMV spreads into the lungs following fetal viremia. Usual pathological findings in congenitally infected fetal lungs are immature and dystelectatic lungs associated with interstitial oedema, the absence of cellular reaction, or purulent inflammation [10, 11]. Vasculitis is another suspected pathogenic mechanism leading to neonatal demise [12, 13]. In allogenic transplant recipients, CMV pneumonitis was described as an immunopathogenic mechanism due to a T-cell response to viral antigens expressed on lung cells [14]. However, a case of extensive CMV infection presented with severe pulmonary hypertension has been reported in an adult with acquired immune deficiency syndrome [15]. Post-mortem examination showed infection in the lung endothelial cells. Enlarged CMV-infected endothelial cells protruded into and compromised the lumens of the small vessels, leading to a significant increase in pulmonary arterial resistances. According to these pathologic features, pulmonary hypertension may be transient if specific antiviral therapy is initiated. Respiratory support including mechanical ventilation, NO, and ECMO should be initiated and maintained as long as pulmonary hypertension persists. These measures may help to control respiratory status as long as antiviral therapy may be effective.

Antiviral treatment options are limited. Ganciclovir was first used in children over 20 years ago, but the optimal dose, duration, and route of administration remain poorly evidence based [6]. The benefits of early treatment with ganciclovir in congenital CMV infection, especially on sensorineural hearing loss, remain controversial [16, 17]. However, congenitally infected infants with severe organ disease such as pneumonitis, oesophagitis, persistent severe thrombocytopenia, and active sight-threatening chorioretinitis appeared to benefit from ganciclovir therapy [5, 18]. In the first case reported here, respiratory status was severely affected when ganciclovir was initiated. This precluded expecting a significant improvement. In the second case, when ganciclovir therapy was associated with respiratory support, respiratory status improved and the patient recovered. We did not observe any side effect although ganciclovir-related severe but reversible neutropenia has been reported in two-thirds of the treated infants [16]. This suggests that the administration of antiviral therapy is appropriate in severe condition attributed to CMV infection. However, larger studies should be designed to drive further evidence on clinical benefit of this treatment.

Polyclonal globulins in association with ganciclovir were used in the first case. Specific anti-CMV immunoglobulins are not available in France. CMV immunoglobulin therapy has not been directly evaluated for the treatment of neonates with symptomatic congenital CMV disease, but it may be effective in case of transfusion-acquired CMV infection [18]. The benefit of using CMV immunoglobulins has been evaluated in the prevention of CMV disease after lung transplantation but failed to improve outcome [19]. In CMV seropositive autologous hematopoietic stem cell recipients, the outcome of CMV pneumonia remains poor despite treatment with ganciclovir and CMV hyperimmune globulin administration [20].

Preconceptional immunity to CMV provides incomplete protection, and congenital CMV infection can occur in infants following both primary and recurrent infection in the mother [21]. Although the latter is less prevalent, the prognosis of fetal infection is similar to that following primary infection [3, 4, 21]. Recurrent infection is caused either by reinfection with a new CMV strain or reactivation of a latent infection [18]. Reactivation of CMV infection during pregnancy is reported in 10–30% of seropositive women, and the risk of transmitting the virus is about 1–3% [22]. In the second case, we reported CMV-positive urine on the first day of life excluded a postnatal CMV infection. In case of maternal recurrent infection, we can hypothesize that maternal antibodies in association with antiviral therapy may help to recovery.

## 3. Conclusion

This report highlights the potential implication of congenital CMV in severe PPH, even in case of recurrent maternal CMV infection. PPH should be systematically investigated by echocardiography in cases of congenital CMV pneumonitis. Severe neonatal PPH with no obvious aetiology should imply searching for congenital CMV infection. In cases with life-threatening PPH, the administration of antiviral therapy appears appropriate, associated with respiratory support including extracorporeal ventilation, as long as severe neurological impairment has been excluded. The severity of the clinical condition and progressive end-organ disease outweigh the potential toxicity of this treatment.
